# Integrative Bulk and Single-Cell Transcriptomic Analysis Identifies a Hypoxia- and Lipid Metabolism-Related Prognostic Signature in Oral Squamous Cell Carcinoma: A Retrospective Study

**DOI:** 10.3390/ijms27104564

**Published:** 2026-05-19

**Authors:** Li Zhao, Jiale Wang, Kaiyuan Jiang, Kun Wang, Linglin Zhang

**Affiliations:** State Key Laboratory of Oral Diseases & National Center for Stomatology & National Clinical Research Center for Oral Diseases, West China Hospital of Stomatology, Sichuan University, Chengdu 610041, China; zhaoliiii_1111@163.com (L.Z.); wangjialeshirlly@163.com (J.W.); jiangkaiyuan0701@163.com (K.J.)

**Keywords:** oral squamous cell carcinoma, hypoxia, lipid metabolism, prognostic signature, immune microenvironment, single-cell RNA sequencing

## Abstract

Oral squamous cell carcinoma (OSCC) is a biologically heterogeneous malignancy with poor clinical outcomes. Hypoxia and lipid metabolic reprogramming are important drivers of OSCC progression and treatment adaptation, and these processes are biologically interconnected. However, prognostic studies integrating hypoxia- and lipid metabolism-related features in OSCC remain limited. Here, transcriptomic data from TCGA-HNSC-OSCC were integrated with curated hypoxia- and lipid metabolism-related genes to identify candidate genes, construct a prognostic signature, and characterize its biological relevance through enrichment analysis, immune profiling, single-cell RNA-sequencing analysis, and RT-qPCR validation. A four-gene signature consisting of STC2, CAV1, ACADL, and PLA2G2D showed stable prognostic performance in the TCGA-HNSC-OSCC cohort and the external validation cohort GSE41613. The risk signature remained significantly associated with overall survival after adjustment for clinicopathological factors and retained prognostic discrimination across stage- and nodal status-defined subgroups. The high- and low-risk groups displayed distinct pathway, immune, mutational, and predicted drug sensitivity features. Notably, PLA2G2D showed the strongest association with differential immune infiltration, whereas single-cell analysis identified endothelial cells as a major CAV1-enriched population with active intercellular communication and dynamic state transitions. These findings define a hypoxia- and lipid metabolism-related prognostic signature and support its relevance to immune remodeling and endothelial cell context in OSCC.

## 1. Introduction

Oral squamous cell carcinoma (OSCC) arises from the oral mucosal epithelium and accounts for more than 90% of oral malignancies. Despite advances in diagnosis and multidisciplinary treatment, OSCC remains a major clinical challenge because of its aggressive local invasion, frequent cervical lymph node metastasis, high risk of recurrence, and substantial impairment of oral function and quality of life [1,2,3]. Established etiological factors include tobacco exposure, alcohol consumption, and betel quid or areca nut chewing. OSCC may also arise from oral potentially malignant disorders, and human papillomavirus infection has been associated with a subset of OSCC cases [1,4,5]. Current management is still centered on surgery, combined with radiotherapy, chemotherapy, targeted therapy, or immunotherapy according to disease stage and risk profile [6,7]. However, marked molecular heterogeneity and variable therapeutic responses continue to limit durable clinical benefit, highlighting the need for more informative biomarkers and biologically meaningful risk stratification strategies in OSCC [8,9,10].

Among the major microenvironmental pressures involved in OSCC progression, hypoxia and metabolic reprogramming have attracted increasing attention. Hypoxia is a fundamental feature of solid tumors and activates hypoxia-inducible factor (HIF)-dependent transcriptional programs that reshape angiogenesis, epithelial plasticity, immune regulation, and treatment response [11,12]. In OSCC, elevated HIF-1α expression has been associated with aggressive clinicopathological characteristics and unfavorable survival outcomes [13,14]. In parallel, hypoxia-related gene signatures have shown prognostic relevance and links with immune infiltration patterns in OSCC [15,16,17]. Lipid metabolic reprogramming represents another important component of malignant progression. Altered lipid uptake, synthesis, storage, desaturation, and utilization provide structural lipids, energy sources, and signaling mediators that support tumor growth, stress adaptation, and tumor–stroma communication [18,19]. Recent studies in oral cancer further indicate that disturbed lipid homeostasis contributes to tumor progression and may reveal potential biomarkers and therapeutic vulnerabilities [20,21,22].

Importantly, hypoxia and lipid metabolism are not independent events but closely interconnected adaptive processes. Under oxygen-limited conditions, cancer cells can remodel multiple lipid metabolic pathways to maintain membrane and redox homeostasis and to sustain survival under environmental stress [23,24]. In OSCC, stromal lipid support has also emerged as a relevant mechanism. Enhanced lipid biosynthesis in cancer-associated fibroblasts promotes tumor progression through the IL8/AKT/p-ACLY axis, while cancer-associated fibroblast (CAF)-derived fatty acids can facilitate lipid raft formation and activate PI3K/AKT signaling in OSCC cells [25,26]. Although hypoxia-related signatures and lipid metabolism-associated alterations have each been reported in OSCC, these two biological dimensions have more often been investigated separately. Their integrated prognostic significance, together with potential associations with the immune landscape and cellular context, remains insufficiently characterized. In the present study, transcriptomic data from TCGA-HNSC-OSCC were integrated with curated hypoxia- and lipid metabolism-related gene sets to identify prognostic candidates and construct a risk signature. Functional enrichment analysis, immune infiltration profiling, and single-cell transcriptomic analysis were further incorporated to define the biological relevance and cellular context of these genes, followed by experimental validation of key expression patterns. This integrative analysis identified a hypoxia- and lipid metabolism-related prognostic signature, thereby providing a useful framework for prognostic stratification and microenvironment-oriented investigation in OSCC.

## 2. Results

### 2.1. Identification of Hypoxia- and Lipid Metabolism-Related Candidate Genes (CGs) in Oral Squamous Cell Carcinoma (OSCC)

**Figure 1:**
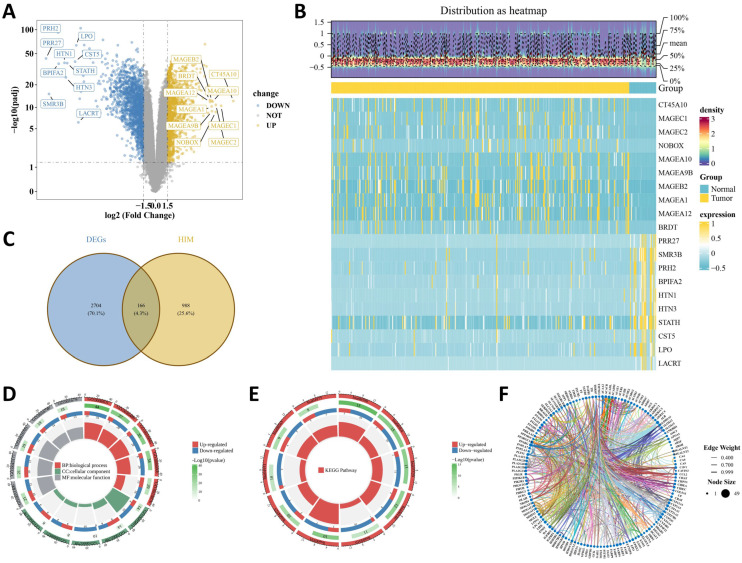
Identification of hypoxia- and lipid metabolism-related candidate genes in OSCC. (**A**) Volcano plot showing differentially expressed genes (DEGs) between tumor and normal samples in the TCGA-HNSC-OSCC cohort. (**B**) Heatmap showing the top 10 upregulated and top 10 downregulated DEGs. (**C**) Venn diagram showing the overlap between DEGs and hypoxia- and lipid metabolism-related genes (HLMRGs). (**D**) Gene Ontology (GO) enrichment analysis of candidate genes (CGs). (**E**) Kyoto Encyclopedia of Genes and Genomes (KEGG) enrichment analysis of CGs. (**F**) Protein–protein interaction (PPI) network of CGs. Each node represents a protein, and each edge represents a predicted interaction.

Differential expression analysis of the TCGA-HNSC-OSCC cohort identified 2870 differentially expressed genes (DEGs), including representative upregulated genes such as MAGEB2 and CT45A10 and representative downregulated genes such as PRH2, HTN1, STATH, LPO, and LACRT (Figure 1A,B; Table S3). Intersecting these DEGs with hypoxia- and lipid metabolism-related genes (HLMRGs) yielded 166 CGs (Figure 1C; Table S4). Functional enrichment analysis showed that these CGs were highly converged on lipid-associated biological programs. The top Gene Ontology (GO) biological process terms included fatty acid metabolic process, glycerolipid metabolic process, and lipid catabolic process, whereas the enriched cellular component terms highlighted lipid droplet and endoplasmic reticulum/peroxisome-related compartments. Molecular function terms were mainly associated with carboxylic acid binding and oxidoreductase-related activities (Figure 1D, Table S5). Kyoto Encyclopedia of Genes and Genomes (KEGG) analysis further linked the CGs to arachidonic acid metabolism, glycerophospholipid metabolism, and the PPAR signaling pathway (Figure 1E; Table S6). Notably, several core enriched terms contained more downregulated than upregulated genes, suggesting coordinated remodeling rather than uniform activation of lipid metabolism. The protein–protein interaction (PPI) network showed a densely connected interaction pattern, with PPARG and several metabolism-related genes occupying relatively central positions, supporting subsequent prioritization of key genes for prognostic modeling and downstream characterization (Figure 1F).

### 2.2. Construction and Validation of a Four-Gene Hypoxia- and Lipid Metabolism-Related Prognostic Signature

**Figure 2:**
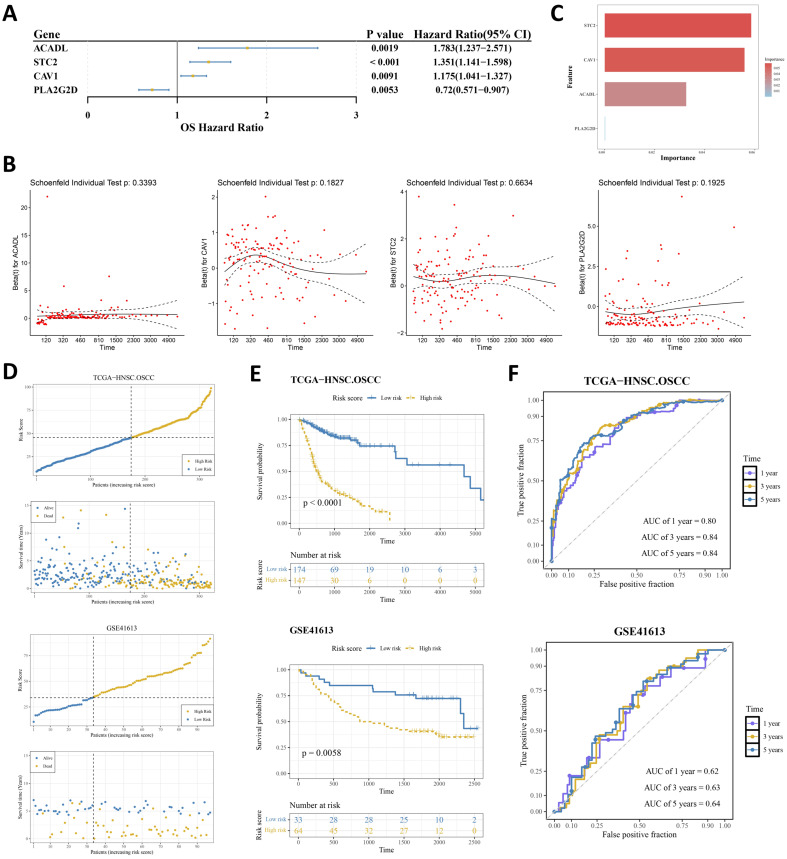
Construction and validation of a four-gene hypoxia- and lipid metabolism-related prognostic signature. (**A**) Forest plot of univariate Cox regression analysis of prognostic genes. (**B**) Proportional hazards (PH) test of the prognostic genes. Red dots represent Schoenfeld residuals for each subject at the corresponding censoring time. The solid line indicates the smoothed fit curve, reflecting the overall temporal trend of residuals. The dashed lines represent the 95% confidence interval of the smoothed curve. (**C**) Variable importance of the prognostic genes in the RSF model. (**D**) Risk score distribution and survival status of patients in the TCGA-HNSC-OSCC training cohort and the GSE41613 validation cohort. (**E**) K–M survival curves for the high-risk and low-risk groups in the TCGA-HNSC-OSCC and GSE41613 cohorts. (**F**) Time-dependent ROC curves for predicting 1-, 3-, and 5-year overall survival in the TCGA-HNSC-OSCC and GSE41613 cohorts.

Univariate Cox regression analysis identified four prognostic genes among the hypoxia- and lipid metabolism-related CGs that satisfied both the significance threshold (*p* < 0.01, HR ≠ 1) and the proportional hazards (PH) assumption (*p* > 0.05), namely ACADL, STC2, CAV1, and PLA2G2D. Among them, PLA2G2D was associated with decreased mortality risk, whereas the others showed an opposite association (Figure 2A,B; Table S7). In the random survival forest (RSF) model, STC2 showed the highest variable importance, followed by CAV1, ACADL, and PLA2G2D (Figure 2C).

Based on the optimal cutoff, Using the optimal cutoff values, patients in the TCGA-HNSC-OSCC cohort were divided into a high-risk group of 147 cases and a low-risk group of 174 cases (Table S8), whereas patients in the GSE41613 cohort were divided into a high-risk group of 64 cases and a low-risk group of 33 cases (Table S9). In both cohorts, the high-risk group showed higher risk scores and poorer survival outcomes than the low-risk group (Figure 2D). Kaplan–Meier analysis further showed significantly poorer overall survival in the high-risk group in both the training and validation cohorts (*p* < 0.05) (Figure 2E). Time-dependent ROC analysis demonstrated good discrimination in the training cohort (AUC > 0.8) and moderate but reproducible predictive ability in the external validation cohort (AUC > 0.6), indicating that the four-gene signature had stable risk stratification value across datasets (Figure 2F).

### 2.3. Baseline Characteristics According to Risk Stratification

After establishing the four-gene risk classification, baseline clinicopathological variables and available etiological annotations were summarized to characterize the clinical context of the high- and low-risk groups (Table 1). The high-risk group contained higher proportions of patients with advanced T stage, nodal involvement, and advanced overall stage than the low-risk group. In contrast, age, gender, and M stage did not differ significantly between the two groups. Regarding available etiological annotations, current or former smoking was more frequent in the high-risk group than in the low-risk group (*p* = 0.014). A similar pattern was observed for drinking history (*p* = 0.006). For betel nut use, the available TCGA-HNSC annotation did not distinguish positive from negative exposure status and was missing in most patients. Therefore, this variable was summarized descriptively and was not used for formal exposure-based comparison. HPV status was unavailable for all patients in the TCGA-HNSC-OSCC subset analyzed here, precluding HPV-stratified survival analysis.

### 2.4. Independent Prognostic Value and Subgroup Stratification of the Risk Signature

**Figure 3:**
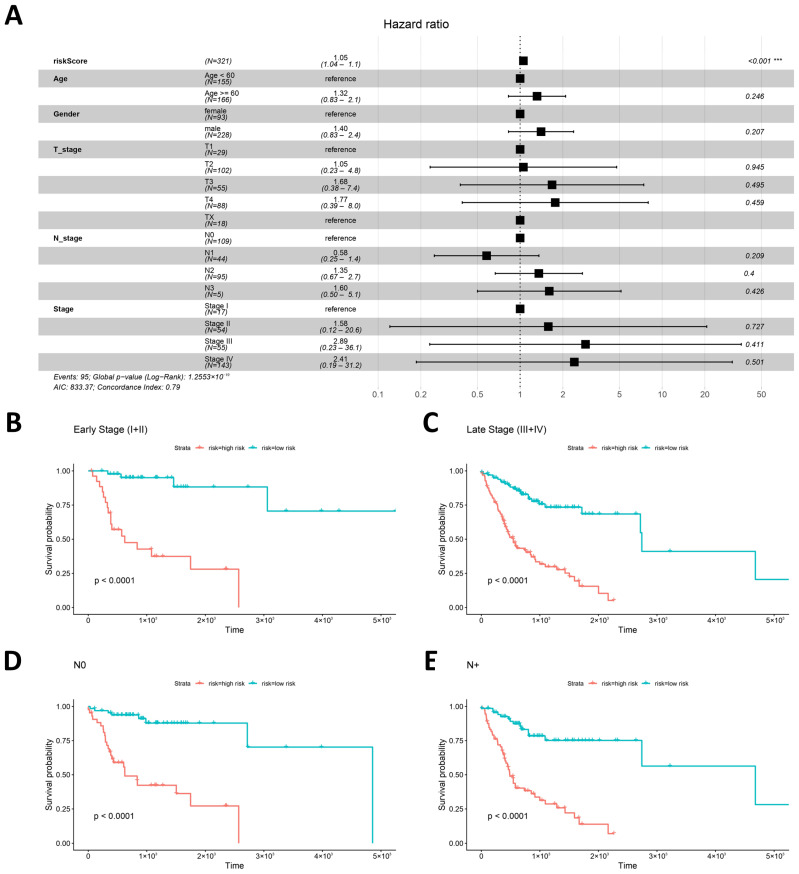
Independent prognostic value and subgroup stratification of the hypoxia- and lipid metabo-lism-related risk signature. (**A**) Forest plot of multivariable Cox regression analysis incorporating risk score, age, gender, T stage, N stage, and overall stage. (**B**–**E**) Kaplan–Meier survival curves comparing overall survival between the high-risk and low-risk groups in clinically stratified sub-groups: (**B**) early-stage patients (stage I–II), (**C**) advanced-stage patients (stage III–IV), (**D**) node-negative patients (N0), and (**E**) node-positive patients (N+). *** *p* < 0.001.

Having characterized the baseline features of the high- and low-risk groups, the prognostic robustness of the risk signature was further evaluated in relation to conventional clinicopathological variables. Multivariable Cox regression analysis was performed by incorporating the risk score, age, gender, T stage, N stage, and overall stage (Figure 3A). The risk score remained significantly associated with unfavorable overall survival after adjustment for these covariates (HR = 1.05, 95% CI: 1.04–1.10, *p* < 0.001). This finding suggested that the hypoxia- and lipid metabolism-related risk signature may provide prognostic information complementary to conventional clinical staging parameters.

Stratified Kaplan–Meier survival analyses were then performed to determine whether the prognostic value of the signature was maintained within clinically relevant subgroups defined by overall stage and nodal status. The high-risk group showed significantly poorer overall survival than the low-risk group among patients with early-stage disease (stage I–II), advanced-stage disease (stage III–IV), node-negative disease (N0), and node-positive disease (N+) (all log-rank *p* < 0.0001, Figure 3B–E). These results indicated that the four-gene signature retained prognostic discrimination across stage- and nodal status-defined subgroups, supporting its potential value as an adjunctive molecular layer for refining risk stratification in OSCC.

### 2.5. Pathway Characteristics Associated with the Prognostic Genes and Risk Stratification

**Figure 4:**
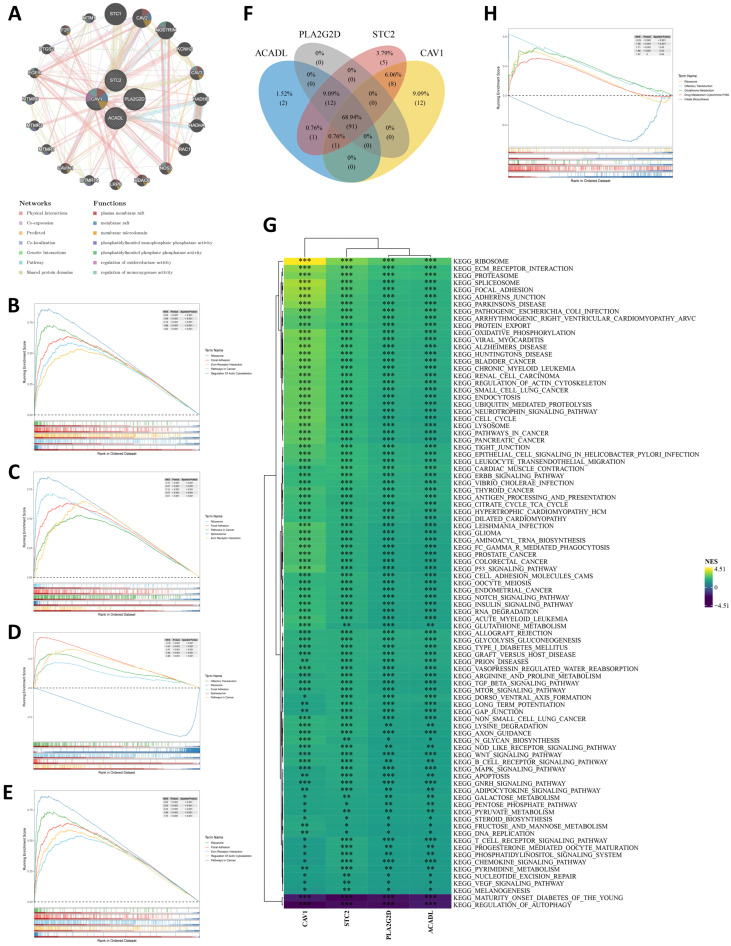
Pathway characteristics associated with the prognostic genes and risk stratification. (**A**) GeneMANIA network of the four prognostic genes and their functionally related genes. Top five enriched pathways associated with (**B**) ACADL, (**C**) STC2, (**D**) CAV1, and (**E**) PLA2G2D. (**F**) Venn diagram showing the common pathways shared by the four prognostic genes. (**G**) Heatmap showing the correlations between the prognostic genes and the shared pathways. (**H**) Top five differentially enriched pathways between the high-risk and low-risk groups. * *p* < 0.05, ** *p* < 0.01, *** *p* < 0.001.

The interaction network and pathway landscape of the four prognostic genes were first characterized. GeneMANIA analysis showed that the four prognostic genes were connected with genes such as STC1, CAV2, NOSTRIN, HADHA, HADHB, NOS3, CAVIN1, EGFR, and PTGS2, and the main functional annotations were enriched in plasma membrane raft or membrane microdomain and regulation of oxidoreductase activity (Figure 4A). Gene-level GSEA showed that the four prognostic genes were recurrently associated with ribosome, focal adhesion, ECM–receptor interaction, pathways in cancer, regulation of actin cytoskeleton, and spliceosome-related programs (Figure 4B–E), indicating convergence on translation-related and adhesion/cytoskeleton-remodeling processes rather than a single biological axis. Moreover, a total of 91 common pathways were shared by the four prognostic genes (Figure 4F). The shared-pathway NES heatmap further indicated that ribosome was positively enriched across the four genes, most prominently in CAV1, whereas regulation of autophagy showed negative enrichment (Figure 4G; Table S10). These common pathways spanned multiple biological dimensions, including metabolic, immune, signaling, and cancer-related processes. GSEA between the high- and low-risk groups revealed differential enrichment of ribosome, glutathione metabolism, drug metabolism–cytochrome P450, and folate biosynthesis, suggesting that risk stratification was accompanied by coordinated differences in translational and redox/metabolic states (Figure 4H; Table S11). Taken together, these results indicate that the prognostic signature was associated with a pathway landscape centered on protein translation, cell adhesion and cytoskeleton remodeling, and metabolic stress responses in OSCC.

### 2.6. Immune Landscape Associated with Risk Stratification

**Figure 5:**
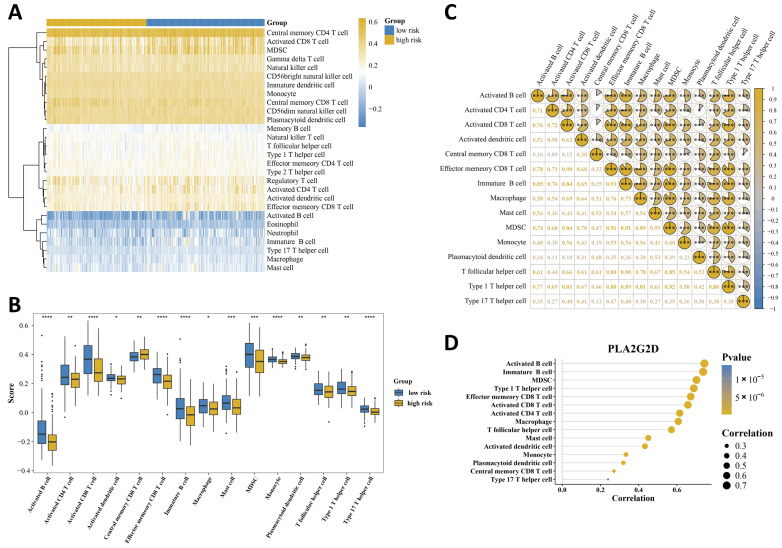
Immune landscape associated with risk stratification. (**A**) Relative proportions of 28 immune cell types in the high-risk and low-risk groups. (**B**) Differentially infiltrated immune cell types between the high-risk and low-risk groups. (**C**) Correlation matrix of differential immune cell populations. Circle size and color intensity indicate correlation strength (r); yellow denotes positive and blue denotes negative correlations. (**D**) Correlations between PLA2G2D expression and differential immune cell populations. * *p* < 0.05, ** *p* < 0.01, *** *p* < 0.001, **** *p* < 0.0001.

Immune deconvolution analysis showed that the low-risk group exhibited higher infiltration levels of multiple activated lymphoid and myeloid populations, including activated B cells, activated CD4 T cells, activated CD8 T cells, activated dendritic cells, effector memory CD8 T cells, immature B cells, macrophages, myeloid-derived suppressor cells (MDSCs), monocytes, plasmacytoid dendritic cells, T follicular helper cells, type 1 T helper cells, and type 17 T helper cells, whereas central memory CD8 T cells were relatively higher in the high-risk group (Figure 5A,B). These findings indicated that the low-risk group was characterized by a more immune-infiltrated microenvironment. The correlation analysis further showed that these immune cell subsets varied in a coordinated manner rather than isolate variation (Figure 5C). Notably, activated B cells, immature B cells, T follicular helper cells, activated CD8 T cells, and effector memory CD8 T cells displayed concordant positive correlations, suggesting coordinated enrichment of an adaptive immune cell cluster in the low-risk group. Among the four prognostic genes, PLA2G2D showed the strongest and broadest positive association with immune infiltration, especially with activated B cells, immature B cells, MDSCs, type 1 T helper cells, effector memory CD8 T cells, and activated CD8 T cells (Figure 5D; Table S12). Together, these findings highlighted PLA2G2D as the prognostic gene most closely linked to the immune-infiltrated phenotype in OSCC.

### 2.7. Genomic, Therapeutic, and Clinicopathological Associations of Risk Stratification

Somatic mutation analysis showed distinct somatic mutation landscapes between the high- and low-risk groups, although both groups retained the TP53-dominant and missense mutation-predominant pattern typical of OSCC (Figure 5A,B). The high-risk group showed a higher overall alteration rate and a higher frequency of TP53 mutations. The ESTIMATE analysis further showed that the high-risk group had significantly lower immune and ESTIMATE scores, while no significant difference was observed in stromal score (Figure 5C), suggesting a less immune-enriched tumor microenvironment. Consistently, 27 immune checkpoint-related genes were differentially expressed between the two groups, with CD276 upregulated and multiple lymphocyte-associated checkpoints or costimulatory genes, such as TNFRSF18, CTLA4, ICOS, PDCD1, and CD27, downregulated in the high-risk group (Figure 5D).

**Figure 6:**
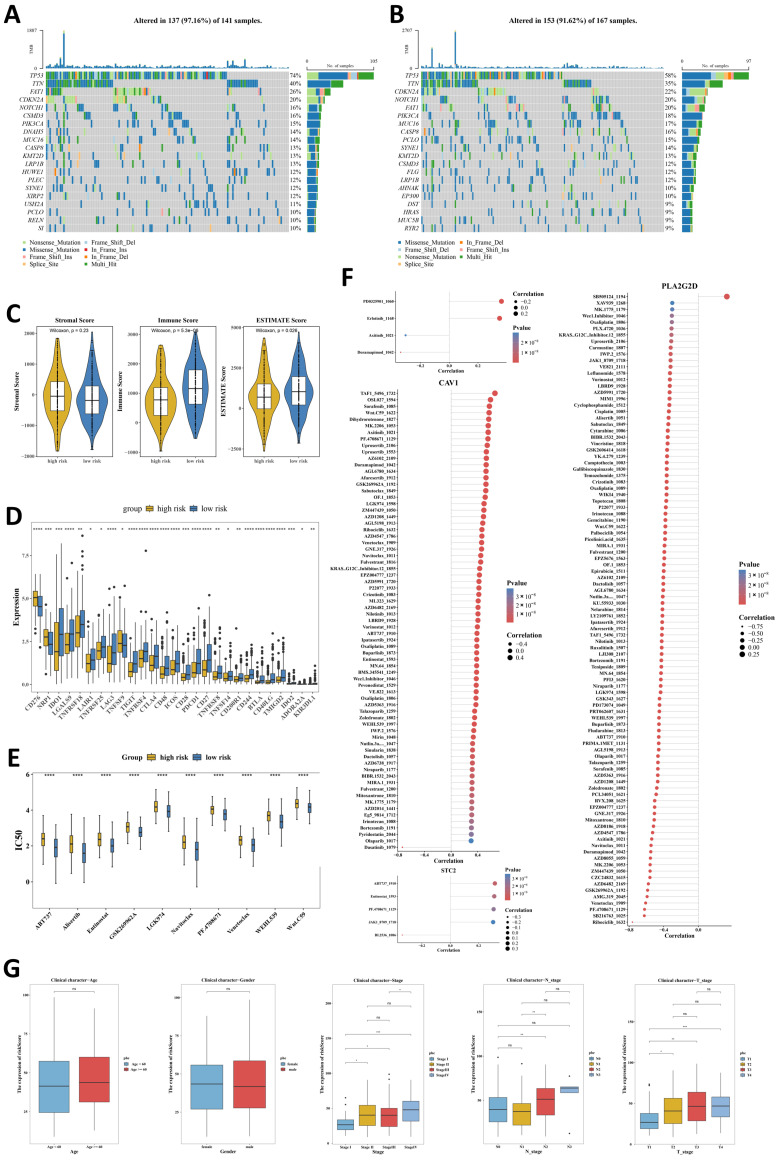
Genomic, therapeutic, and clinicopathological associations of risk stratification. Somatic mutation profiles of the (**A**) high-risk group and (**B**) low-risk group. (**C**) Comparison of immune score, stromal score, and ESTIMATE score between the high-risk and low-risk groups. (**D**) Differential expression of immune checkpoint-related genes between the high-risk and low-risk groups. (**E**) Differential predicted IC50 values between the high-risk and low-risk groups. (**F**) Correlations between prognostic genes and drugs showing differential IC50 values. (**G**) Associations between risk scores and clinicopathological features. ns, not significant, * *p* < 0.05, ** *p* < 0.01, *** *p* < 0.001, **** *p* < 0.0001.

Drug sensitivity analysis identified 149 compounds with differential predicted IC50 values between the two groups, and the representative compounds consistently exhibited higher IC50 values in the high-risk group (Figure 6E; Table S13). Correlation analysis further revealed distinct gene-specific drug-response patterns, with CAV1 showing the strongest positive association with TAF1_5496_1732, whereas PLA2G2D displayed broad negative correlations with multiple compounds, including a marked inverse association with Ribociclib_1632 (Figure 6F; Table S14). In addition, higher risk scores were associated with more advanced overall stage, N stage, and T stage, whereas no significant associations were observed with age or gender (Figure 6G). Taken together, these findings indicate that the prognostic signature was associated with distinct mutational features, immune contexture, predicted therapeutic sensitivity, and disease progression in OSCC.

### 2.8. Identification of Endothelial Cells as a Major CAV1-Enriched Cell Population in OSCC Through Single-Cell Transcriptomic Analysis

**Figure 7:**
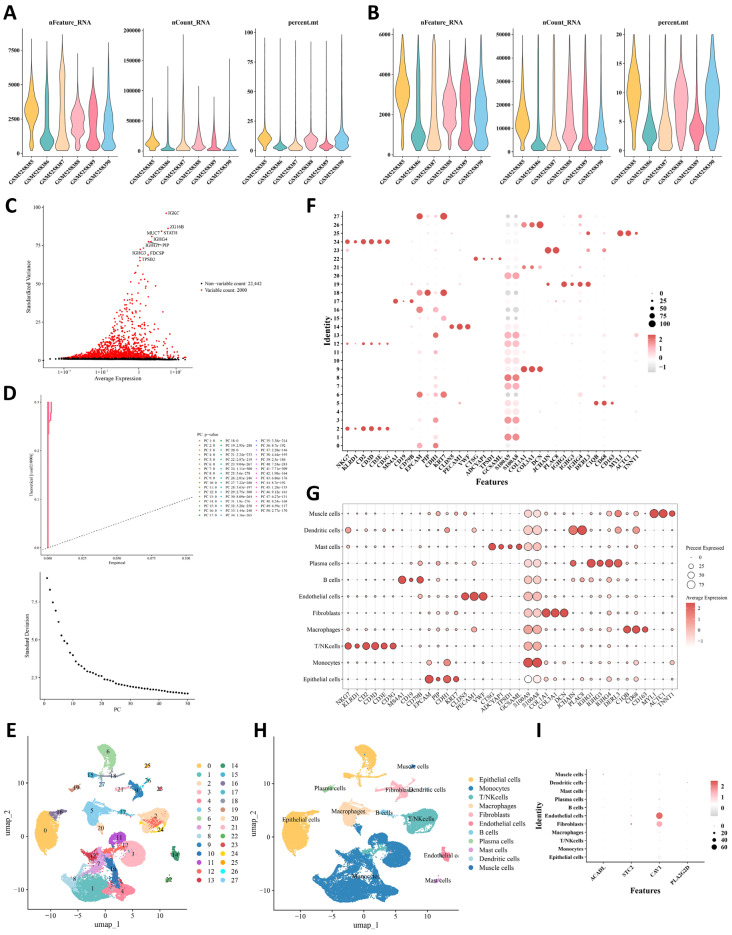
Identification of endothelial cells as a major CAV1-enriched cell population in OSCC through single-cell transcriptomic analysis. The number of detected genes (nFeature_RNA), total counts (nCount_RNA), and mitochondrial gene proportion (percent.mt) (**A**) before and (**B**) after quality control. (**C**) Highly variable genes used for downstream single-cell analysis. (**D**) Principal component analysis of single cells. Due to overlap between groups, colors may partially overlap. (**E**) UMAP plot showing the cell clusters. Expression patterns of marker genes across (**F**) cell clusters and (**G**) annotated cell types. (**H**) UMAP plot showing the annotated cell types. (**I**) Expression patterns of the prognostic genes across the annotated cell populations.

After quality control, 54,980 cells with 24,442 detected genes were retained for downstream analyses (Figure 7A,B). Based on the highly variable genes and principal component analysis, cells were subjected to unsupervised clustering and resolved into 28 clusters (Figure 7C–E). Marker-based annotation further grouped these clusters into 11 major cell types, including epithelial cells, monocytes, T/NK cells, macrophages, fibroblasts, endothelial cells, B cells, plasma cells, mast cells, dendritic cells, and muscle cells (Figure 7F–H). Canonical markers supported the annotations, such as EPCAM/CDH1/KRT7 for epithelial cells, S100A8/S100A9 for monocytes, NKG7/KLRD1/CD3D for T/NK cells, COL1A1/COL3A1/DCN for fibroblasts, and CLDN5/PECAM1/VWF for endothelial cells. Among the four prognostic genes, CAV1 showed the most prominent and concentrated expression in endothelial cells, with only limited signal in fibroblasts and epithelial cells, whereas ACADL, STC2, and PLA2G2D were sparsely expressed across annotated cell types (Figure 7I). These findings identified endothelial cells as the predominant CAV1-expressing cell type in OSCC and provided a cellular basis for subsequent analyses of their functional states and microenvironmental interactions.

### 2.9. Characterization of Endothelial Cell Communication, Trajectory, and Metabolic Features in OSCC

To further characterize the endothelial compartment highlighted by single-cell analysis, cell–cell communication, pseudotime trajectory, and functional features of endothelial cells were subsequently investigated.

**Figure 8:**
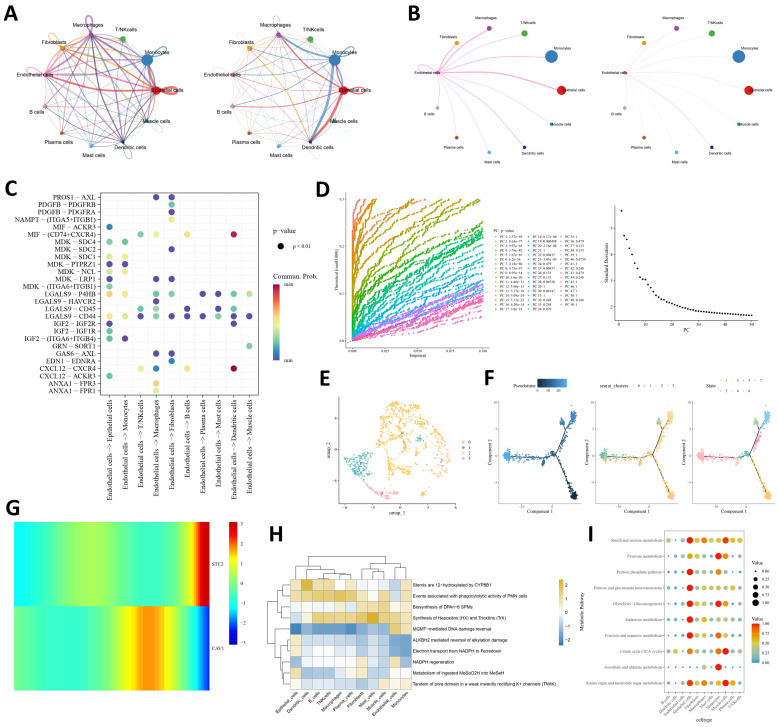
Characterization of endothelial cell communication, trajectory, and metabolic features in OSCC. (**A**) CellChat network showing the number and strength of interactions among major cell types. (**B**) CellChat network showing the number and strength of interactions between endothelial cells and other cell types. (**C**) Bubble plot showing representative ligand–receptor interactions among cell types. (**D**) Principal component analysis of endothelial cells. (**E**) UMAP plot showing the re-clustered endothelial subpopulations. (**F**) Pseudotime analysis of endothelial cells showing the differentiation trajectory by pseudotime state (**left**), endothelial subclusters (**middle**), and developmental stage (**right**). (**G**) Heatmap showing the expression dynamics of the prognostic genes along the endothelial trajectory. (**H**) Functional enrichment analysis of endothelial cells. (**I**) Metabolic activity profiles of endothelial cells.

Cell–cell communication analysis showed that epithelial cells and monocytes acted as major interaction hubs in the OSCC microenvironment, whereas endothelial cells maintained broad outgoing signaling toward multiple cell types (Figure 8A,B). When endothelial cells were examined specifically as sender cells, they displayed the largest number of inferred interactions with epithelial cells, whereas the strongest communication weights were directed toward dendritic cells and monocytes (Figure 8C). Among the ligand–receptor pairs, CXCL12–CXCR4 showed the highest communication probability, particularly in endothelial cell interactions with dendritic cells, highlighting a potential role of endothelial cells in shaping local immune communication (Figure 8C). Re-clustering of endothelial cells identified four subclusters (Figure 8D,E).

Pseudotime analysis further revealed a branched trajectory, with cluster 1 enriched at the early stage, cluster 0 distributed along the main trajectory, cluster 2 concentrated in a terminal branch, and cluster 3 restricted to a minor branch (Figure 7F). Along this trajectory, CAV1 was relatively elevated at an intermediate stage, whereas STC2 increased toward the late stage (Figure 7G). Functional analyses further suggested that endothelial cells were relatively enriched in lipid mediator-related processes such as hepoxilin and trioxilin synthesis, while exhibiting overall low metabolic activity across major carbohydrate pathways, with only modest glycolytic signals retained (Figure 7H,I). Together, these findings indicate that endothelial cells in OSCC represent a communication-active but metabolically restrained compartment with dynamic state transitions.

### 2.10. Expression Pattern of Prognostic Genes in OSCC

**Figure 9:**
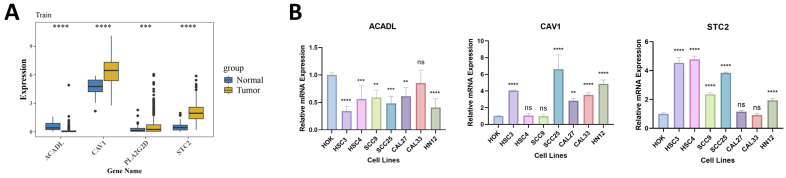
Expression patterns of the prognostic genes in OSCC. (**A**) Differential expression of the prognostic genes in the TCGA-HNSC-OSCC cohort. (**B**) RT-qPCR validation of prognostic gene expression in OSCC cell lines relative to HOK cells. PLA2G2D transcripts were not reliably detected in the tested cell lines. ns, not significant, ** *p* < 0.01, *** *p* < 0.001, **** *p* < 0.0001.

The differential expression analysis in TCGA-HNSC-OSCC showed that STC2, CAV1, and PLA2G2D were upregulated in the tumor tissues, while ACADL was downregulated (Figure 9A). RT-qPCR further evaluated the expression of these prognostic genes in OSCC cell lines using HOK cells as the normal control. Overall, STC2 showed an increasing trend in most OSCC cell lines, whereas ACADL was generally decreased. CAV1 displayed a more heterogeneous expression pattern, although an overall increasing trend was still observed in most OSCC cell lines (Figure 9B; Table S15). Thus, the in vitro results largely supported the expression trends of STC2 and ACADL, while suggesting that the expression of CAV1 may be more context-dependent across different OSCC cell lines. In contrast, PLA2G2D transcripts were not reliably detected in the tested cell lines, precluding robust quantitative comparison by RT-qPCR. This finding may reflect the extremely low abundance of PLA2G2D in isolated epithelial cell models and is consistent with its closer association with the immune microenvironment than with tumor cell-intrinsic expression.

## 3. Discussion

The present study identifies a four-gene signature related to hypoxia and lipid metabolism that supports prognostic stratification in OSCC and also provides a biologically meaningful framework for interpreting tumor heterogeneity. Within the current level of evidence, this model is best considered a tissue-based molecular biomarker for biological subclassification and risk estimation in OSCC, with additional value for inferring differences in immune and vascular microenvironmental states. This point is important in the context of oral disease biomarker discovery, because prognosis in OSCC is still constrained by the limited biological resolution of conventional clinicopathological classification alone [1,3,27]. By integrating transcriptomic risk modeling, immune analysis, pathway analysis, and single-cell interpretation, the present work suggests that this signature reflects an organized disease state linked to stress adaptation, immune remodeling, and endothelial context. In clinical terms, such a model may help refine risk assessment and generate hypotheses for more personalized follow-up and therapeutic stratification, especially if future validation confirms its reproducibility in clinically deployable tissue-based assays.

From a clinical perspective, the proposed signature should be interpreted as a potential adjunct to established clinicopathological risk assessment rather than as a standalone decision-making tool. Current OSCC management is primarily guided by tumor extent, nodal status, resectability, and postoperative pathological risk factors, including margin status, extranodal extension, depth of invasion, perineural invasion, lympho-vascular invasion, and nodal burden [3,28,29]. In the present study, the risk score remained significantly associated with overall survival after adjustment for available clinicopathological variables and retained prognostic separation within stage- and nodal status-defined subgroups. These findings suggest that the signature may provide additional molecular information within the limits of the analyzed variables and may help refine risk stratification when combined with established clinicopathological parameters.

Among the four genes, CAV1 and PLA2G2D provide the clearest connection between the signature and the OSCC microenvironment. CAV1 is closely linked to membrane organization, lipid handling, adhesion associated signaling, and vascular biology [28,29,30]. In advanced cancers, elevated CAV1 expression has frequently been associated with invasive progression, metastatic behavior, and treatment adaptation [31]. In OSCC, prior studies have connected CAV1 with aggressive clinicopathological features, poorer outcomes, and altered cisplatin responsiveness, with recent work also implicating ferroptosis-related regulation [32,33,34]. These observations fit well with the present findings, especially because the single-cell analysis localized CAV1 predominantly to endothelial cells. PLA2G2D adds a complementary dimension that is more closely tied to immune regulation and extracellular lipid biology. As a secreted phospholipase-related molecule, PLA2G2D participates in phospholipid turnover and downstream lipid mediator generation, processes that influence immune communication [35,36,37]. In head and neck squamous cell carcinoma (HNSCC), the available evidence has mainly linked PLA2G2D to prognosis, immune-related pathways, and macrophage-associated microenvironmental remodeling [38]. This supports the interpretation that PLA2G2D may function as a lipid-sensitive immunoregulatory signal within the present model. STC2 and ACADL complete the biological logic of the signature. STC2 is a well-established hypoxia-responsive gene that has been associated with aggressive behavior and poor outcomes in OSCC [39,40,41]. ACADL represents the fatty acid oxidation component of the model, and studies in other tumor types suggest links to tumor-suppressive behavior and immune regulation through Hippo YAP-related signaling [42,43]. Taken together, these four genes appear to function as a coordinated set of markers that connect stress adaptation with lipid-linked microenvironmental organization.

The pathway results further support this interpretation. Enrichment patterns involving ribosome, focal adhesion, ECM–receptor interaction, regulation of actin cytoskeleton, glutathione metabolism, and folate biosynthesis suggest that the risk groups differ across translational control, redox buffering, anabolic support, and matrix-associated signaling. Ribosome-related enrichment is particularly informative because it is compatible with increased dependence on ribosome biogenesis, selective translation, and proteostatic maintenance in tumor cells exposed to persistent hypoxic and metabolic stress [44,45,46]. Glutathione- and folate-related changes are also consistent with reprogrammed antioxidant defense and one carbon-dependent biosynthetic support [47,48]. At the same time, focal adhesion- and ECM-associated pathways fit a phenotype characterized by active cell–matrix communication, stromal interaction, and invasion associated plasticity [49,50]. These findings place the present signature within a broader adaptive landscape in which metabolic stress, extracellular matrix remodeling, and invasive competence are biologically connected. For a biomarker-oriented study, this is relevant because it suggests that the prognostic signal is not simply statistical, but rather anchored in pathway-level features that are coherent with known OSCC biology.

The immune findings add further translational relevance. The low-risk subgroup showed enrichment of activated B cells, immature B cells, T follicular helper cells, activated CD8 T cells, and effector memory CD8 T cells, indicating a structured adaptive immune program. Previous OSCC studies have shown that tertiary lymphoid structures (TLSs) are associated with better survival and higher CD8 positive T cell density [51]. Consistent findings in HNSCC have linked TLS-related programs, and in some contexts B cell rich immune programs, with favorable outcome and stronger local antitumor immunity [52,53,54]. On that basis, the low-risk phenotype observed here is compatible with an immune active state that may include TLS-like organization. This interpretation should still remain measured, because the current study relies on bulk immune deconvolution and correlation analysis without direct spatial confirmation. Within this immune context, PLA2G2D showed the broadest positive association with adaptive immune populations, while also correlating with inferred MDSCs. This pattern may indicate that the present model captures immune ecological complexity in which lymphoid activation and counter regulatory myeloid signals coexist within the same microenvironmental setting [35,55,56,57,58]. This view also aligns with the higher immune and ESTIMATE scores in the low-risk group and with the checkpoint expression pattern observed across risk states. From a biomarker perspective, these features suggest that the signature may help distinguish OSCCs with a more immune accessible environment from those with a more immune-constrained phenotype.

The single-cell analysis adds important biological depth by placing endothelial cells at the center of the microenvironmental interpretation. Tumor endothelial cells are now widely recognized as active regulators of angiogenesis, leukocyte trafficking, vascular permeability, immune exclusion, and treatment response [59,60,61]. They can also present immunomodulatory and metabolically specialized states within the tumor microenvironment. In this context, the endothelial enrichment of CAV1 is particularly meaningful as it supports a vascular interpretation of the signal detected by the model. The observed endothelial communication pattern, including the prominence of the CXCL12 and CXCR4 axis, is also biologically plausible in OSCC, given the established relevance of this pathway to stromal interaction, tumor progression, and lymphatic dissemination [62,63]. When considered together with the communication-active and relatively metabolically restrained endothelial phenotype identified here, these results support the view that part of the prognostic signal may reflect vascular immune coupling. This possibility matters clinically because abnormal tumor vasculature and endothelial dysfunction can limit immune infiltration, reinforce local immune suppression, and reduce therapeutic responsiveness [64,65,66]. Adverse hypoxia and lipid-related signatures may therefore mark a vascular state that is less permissive to effective immune control.

Several limitations should be acknowledged. This study was retrospective and relied mainly on public transcriptomic datasets, although external validation was included. Clinical and etiological annotations were incomplete. Smoking and drinking histories were available only as broad exposure categories, whereas betel nut use was largely incomplete and HPV status was unavailable in the TCGA-HNSC-OSCC subset, precluding betel nut exposure- or HPV-stratified analyses. Although the risk score retained prognostic value after adjustment for available clinicopathological variables, key postoperative pathological factors and adjuvant treatment information were not incorporated into the current model. Therefore, the signature should be regarded as a potential adjunct to established risk assessment, and its clinical utility should be further evaluated in prospectively annotated OSCC cohorts integrating TNM stage, etiological annotations, postoperative pathological risk factors, and treatment information. In addition, the immune infiltration, pathway activity, and cell–cell communication analyses were computationally inferred and should therefore be interpreted as hypothesis-generating. Although RT-qPCR supported the expression patterns of key genes, functional and spatial validation will be needed to clarify the causal roles of CAV1, PLA2G2D, STC2, and ACADL in hypoxia adaptation, lipid remodeling, immune regulation, and endothelial-cell-associated microenvironmental changes. Future studies integrating spatial transcriptomics or multiplex immunostaining, endothelial cell–OSCC co-culture systems, and targeted gene perturbation under hypoxic and lipid-modulating conditions would help determine whether the low-risk immune-active phenotype corresponds to TLS-enriched niches and how endothelial signaling interfaces with adaptive immunity. Overall, the present study supports a close association among hypoxia- and lipid metabolism-related molecular features, immune remodeling, and endothelial-cell-associated microenvironmental states in OSCC, while identifying CAV1 and PLA2G2D as the two most biologically interpretable candidates for further investigation.

## 4. Materials and Methods

### 4.1. Public Datasets and Collection of HLMRGs

The TCGA-HNSC-OSCC dataset was obtained from The Cancer Genome Atlas Program (TCGA) database (https://portal.gdc.cancer.gov/, accessed on 2 February 2026), consisting of 332 samples from tumor tissues and 30 normal samples. Among them, 321 patients with available survival information were included in the prognostic and survival analyses. Clinical annotations including smoking history, alcohol consumption, and betel nut use were extracted from the TCGA-HNSC-OSCC data files. The GSE41613 dataset (GPL570 platform) was acquired from Gene Expression Omnibus (GEO) database (https://www.ncbi.nlm.nih.gov/geo/, accessed on 2 February 2026), and included 97 samples from OSCC samples with survival information. The single-cell RNA sequencing (scRNA-seq) dataset, GSE172577 (GPL24676 platform, accessed on 2 February 2026), was also acquired from the GEO database and contained 6 OSCC samples. In addition, a total of 1154 hypoxia- and lipid metabolism-related genes (HLMRGs) were collected from a previous research [67] (Table S1).

### 4.2. Identification of DEGs and CGs

DEGs between tumor and normal samples in the TCGA-HNSC-OSCC cohort were identified using the DESeq2 package (v1.40.2) [68] with the thresholds of |log2Foldchange (FC)| > 1.5 and adjusted *p* value (adj. P) < 0.05. The top 10 upregulated and top 10 downregulated genes were visualized in a heatmap and volcano plot using the ComplexHeatmap (v2.15.0) and ggplot2 (v3.5.2) packages, respectively. CGs were defined as the intersection between DEGs and HLMRGs using the ggvenn package (v0.1.10).

### 4.3. Functional Enrichment and PPI Analyses

GO and KEGG enrichment analyses were performed for the CGs using the clusterProfiler package (v4.10.1) [69], with *p* < 0.05 considered statistically significant. GO analysis included the biological process (BP), cellular component (CC), and molecular function (MF) categories. The top five significantly enriched GO terms in each category and the top 10 KEGG pathways were visualized according to *p* value ranking. PPI data for the CGs were retrieved from the Search Tool for the Retrieval of Interacting Genes/Proteins (STRING) database (https://cn.string-db.org/, accessed on 3 February 2026) with an interaction score > 0.4, and the interaction network was visualized using the circlize package (v0.4.10).

### 4.4. Screening of Prognostic Genes

Univariate Cox proportional hazards regression was performed for the key CGs using the survival package (v3.8-3, https://CRAN.R-project.org/package=survival, accessed on 3 February 2026). Genes with *p* < 0.05 and hazard ratio (HR) ≠ 1 were considered candidate prognostic genes. The proportional hazards assumption was further evaluated using the cox.zph function, and genes with *p* > 0.05 in the proportional hazards test were retained. The results were visualized using the forestplot package (v2.0.1) [70].

### 4.5. Construction and Validation of Prognostic Model

A random survival forest (RSF) model was established using the randomForestSRC package (v3.4.1) [71] with ntree = 1000 and mtry = 4, based on the CHF formula:

$$h\left(t|x\right)=\frac{1}{B}\sum_{i=1}^{B}h_{i}\left(t|x\right)$$

Note: h (*t|x*) represents prediction of the tree (*i*) for patient (*x*) in time (*t*).

Risk scores for individual patients were calculated based on the cumulative hazard function (CHF) generated by the RSF model. According to the optimal cutoff value, patients in both the TCGA-HNSC-OSCC and GSE41613 cohorts were stratified into high-risk and low-risk groups. Risk score distribution and survival status were visualized for each cohort.

Kaplan–Meier survival analysis was performed using the survminer package (v0.5.0) [72], and differences between groups were compared using the log-rank test (*p* < 0.05). Time-dependent receiver operating characteristic (ROC) curves for 1-, 3-, and 5-year overall survival were generated using the survivalROC package (v1.0.3.1) [73], with Area Under the Curve (AUC) > 0.6.

Multivariable Cox proportional hazards regression was performed using the survival package (v3.8-3) to assess the independent prognostic value of the risk score. The risk score was included as a continuous variable. Clinicopathological covariates included age dichotomized at 60 years, gender, T stage (T2, T3, and T4 vs. T1), N stage (N1, N2, and N3 vs. N0), and overall stage (II, III, and IV vs. I). The results were visualized as a forest plot using the forestplot package (v2.0.1). For subgroup analysis, patients were stratified by overall stage (I–II vs. III–IV) and nodal status (N0 vs. N+), with N+ defined as N1–N3 disease. The same risk score cutoff defined in the overall TCGA-HNSC-OSCC cohort was applied to all subgroup analyses. Kaplan–Meier survival curves were generated using the survminer package (v0.5.0), and differences in overall survival between the high- and low-risk groups were compared using the log-rank test. *p* < 0.05 was considered statistically significant.

### 4.6. Gene Set Enrichment Analysis (GSEA)

To explore biological pathways associated with the prognostic signature, gene set enrichment analysis (GSEA) was performed using the c2.cp.kegg_legacy.v2025.1.Hs.symbols gene set downloaded from the Molecular Signatures Database (MSigDB) (https://www.gsea-msigdb.org/gsea/msigdb/, accessed on 4 February 2026). Differential expression analysis was conducted on samples with OS data in TCGA-HNSC-OSCC and the DEGs between high-risk and low-risk groups were identified using the DESeq2 package (v 1.40.2) and sorted in descending order of log2FC. GSEA was performed using the ClusterProfiler package (v4.10.1), with |Normalized Enrichment Score (NES)| > 1 and *p* < 0.05 as the significance criteria. The top five enriched pathways were visualized according to *p* value.

To further investigate pathways associated with individual prognostic genes, Spearman correlation analysis between each prognostic gene and all other genes was conducted using the psych package (v2.2.9) [74]. Then, GSEA was performed following the described methods. Commonly enriched pathways among the prognostic genes were summarized using a Venn diagram and heatmap. In addition, the Gene Multiple Association Network Integration Algorithm database (GeneMANIA, https://genemania.org/, accessed on 4 February 2026) was used to explore potential interacting genes and functional associations of the prognostic genes.

### 4.7. Tumor Microenvironment (TME) Analyses

Single-sample gene set enrichment analysis (ssGSEA) was performed using the GSVA package (v1.46.0) [75] to estimate immune cell infiltration in the high-risk and low-risk groups. Differences in immune infiltration were evaluated using the Wilcoxon rank-sum test (*p* < 0.05). In the TCGA-HNSC-OSCC dataset, Spearman correlation analysis was performed using the psych package (v2.2.9) [74] to assess correlations among differentially infiltrated immune cells and between prognostic genes and differentially infiltrated immune cells, with |correlation coefficient (cor)| > 0.3, *p* < 0.05.

Tumor immune dysfunction and exclusion (TIDE) analysis was used to estimate immune dysfunction and exclusion status in the tumor microenvironment, and TIDE scores were compared between the two risk groups using the Wilcoxon rank-sum test (*p* < 0.05). In addition, the expression of immune checkpoint genes reported in a previous study [76] was compared between the high-risk and low-risk groups using the Wilcoxon rank-sum test (*p* < 0.05).

### 4.8. Somatic Mutation Analysis

Somatic mutation profiles of the high-risk and low-risk groups in the TCGA-HNSC-OSCC cohort were analyzed using the maftools package (v2.18.0) [77].

### 4.9. Drug Sensitivity Analysis

Drug sensitivity was predicted for each patient in the TCGA-HNSC-OSCC cohort using the pRRophetic package (v0.5) [78] based on the Genomics of Drug Sensitivity in Cancer (GDSC, https://www.cancerrxgene.org/, accessed on 5 February 2026) database. The estimated half-maximal inhibitory concentration (IC50) values were compared between the high-risk and low-risk groups using the Wilcoxon rank-sum test (*p* < 0.05). Spearman correlation analysis was further performed using the psych package (v2.2.9) [74] to assess the relationships between prognostic gene expression and the IC_50_ values of differentially sensitive drugs, with |cor| > 0.3 and *p* < 0.05 considered significant.

### 4.10. Association Between Risk Score and Clinicopathological Features

Associations between risk score and clinicopathological variables, including age, sex, T stage, N stage, and overall stage, were evaluated using the Wilcoxon rank-sum test (*p* < 0.05). Age was dichotomized at 60 years.

### 4.11. Quality Control of scRNA-seq Data

The GSE172577 scRNA-seq dataset was analyzed using the Seurat package (v5.30) [79]. Cells with 200–4000 detected genes (nFeature_RNA), total read counts of genes (nCount_RNA) < 10000, and mitochondrial gene proportion (percent.mt) < 20% were retained. Genes expressed in at least three cells were included for downstream analysis. Data normalization was performed using the NormalizeData function, and mitochondrial content was calculated using the PercentageFeatureSet function.

### 4.12. Cell Clustering, Annotation, and Identification of Key Cell Populations

The top 2000 genes with the highest variability were identified using the FindVariableFeatures function, and the top 10 most variable genes were visualized by LablePoints function (*p* < 0.05). After data was normalized by Scale Data function, principal component analysis (PCA) was performed for the dimensionality of highly variable genes using the RunPCA function. Based on the *p* value of 1~50 principal components (PCs) calculated through substitution test algorithm of Jackstraw function, we calculated the variance decrease value of each PC using Elbowplot function (*p* < 0.05). The top 30 principal components with the stable standard deviation (SD) were selected for downstream analysis.

Unsupervised clustering was performed using the FindNeighbors and FindClusters functions with a resolution of 0.4. The RunUMAP function was further employed to cluster the cells. Cell clusters were annotated according to marker genes reported in a previous study [80] (Table S2). The expression patterns of the prognostic genes across clusters and annotated cell types were then examined, and cell populations showing differential expression of prognostic genes were defined as key cell populations (*p* < 0.05).

### 4.13. Cell–Cell Communication, Pseudotime, Functional, and Metabolic Analyses of Key Cell Populations

Cell–cell communication between key cell populations and other cell types was analyzed using the CellChat package (v1.6.1) [81], and the number and strength of inferred interactions were visualized.

For pseudotime analysis, key cell populations were re-clustered using the same preprocessing workflow, with the top 20 principal components and a clustering resolution of 0.1. Monocle-based pseudotime analysis was then performed, and trajectories were visualized using the DDRTree package (v 0.1.5, https://CRAN.R-project.org/package=DDRTree, accessed on 5 February 2026). Expression patterns of prognostic genes along the differentiation trajectory were displayed in a heatmap.

Functional pathway analysis of key cell populations involved in OSCC was performed using the analyze_sc_clusters function in the ReactomeGSA package (v1.12.0) [82]. Metabolic pathway activity in key cell populations was assessed using the scMetabolism package (v0.2.1) [82] and the pathways were estimated using the DotPlot.metabolism function.

### 4.14. Cell Culture

Seven OSCC cell lines, including CAL27, CAL33, HSC3, HSC4, SCC9, SCC25, and HN12, were used in this study. Human oral keratinocytes (HOKs) served as normal control cells. All cell lines were generously provided by the State Key Laboratory of Oral Diseases, West China Hospital of Stomatology, Sichuan University (Chengdu, China). CAL27, CAL33, HSC3, HSC4, HN12, and HOK cells were cultured in DMEM (Sigma, Sigma-Aldrich, St. Louis, MO, USA, D5546) supplemented with 10% fetal bovine serum (VivaCell, Shanghai, China, C04001) and 1% penicillin–streptomycin (Gibco, Thermo Fisher Scientific, Waltham, MA, USA, 15070063). SCC9 and SCC25 cells were cultured in DMEM/F12 (Gibco, 11320033) supplemented with 10% fetal bovine serum, 1% penicillin–streptomycin, and hydrocortisone (400 ng/mL; MCE, Monmouth Junction, NJ, USA, HY-N0583). All cells were maintained at 37 °C in a humidified incubator containing 5% CO_2_.

### 4.15. RT-qPCR Validation of Prognostic Gene Expression in OSCC

Expression differences in prognostic genes between tumor and normal tissues in the TCGA-HNSC-OSCC cohort were analyzed using the Wilcoxon rank-sum test (*p* < 0.05). For experimental validation, RT-qPCR was performed in seven OSCC cell lines and one normal control cell line. Total RNA was extracted using the Quick-RNA MiniPrep kit (Zymo Research, Irvine, CA, USA, R1054). Reverse transcription was performed using the PrimeScript FAST RT reagent Kit with gDNA Eraser (Takara, Kusatsu, Shiga, Japan, #RR092A), and quantitative PCR was carried out using TB Green Premix Ex Taq II FAST qPCR (Takara, #CN830A) according to the manufacturer’s instructions. The primer sequences used for RT-qPCR analyses are summarized in Table 2. Relative gene expression was normalized to GAPDH and calculated using the 2^−ΔΔCt^ method. The data were plotted using GraphPad Prism (v9.5) and presented as mean ± standard deviation.

### 4.16. Statistical Analysis

Bioinformatic analyses were performed using R software (v4.3.3). Unless otherwise specified, comparisons between two groups were conducted using the Wilcoxon rank-sum test, and *p* < 0.05 was considered statistically significant. Categorical variables in the baseline characteristics table were compared using the chi-square test or Fisher’s exact test, as appropriate. For RT-qPCR experiments, statistical analysis was performed in GraphPad Prism (v9.5).

## 5. Conclusions

In summary, the hypoxia- and lipid metabolism-related risk model based on ACADL, STC2, CAV1, and PLA2G2D showed potential for prognostic stratification and the prediction of therapeutic sensitivity in OSCC, indicating its value for clinical assessment and more personalized management. Moreover, it may not only expand the range of candidate therapeutic targets for OSCC but also provide new insights into the interplay among hypoxia, lipid metabolism, immune microenvironment remodeling, and tumor progression in OSCC.

## Figures and Tables

**Table 1:** Baseline clinicopathological and etiological characteristics of the high- and low-risk groups in the TCGA-HNSC-OSCC cohort.

Characteristic	All Patients (*N* = 321)	Low-Risk (*n* = 174)	High-Risk (*n* = 147)	*p* Value
Age				0.312
<60 years	118 (36.8)	68 (39.1)	50 (34.0)	
≥60 years	203 (63.2)	106 (60.9)	97 (66.0)	
Gender				0.895
Male	229 (71.3)	124 (71.3)	105 (71.4)	
Female	92 (28.7)	50 (28.7)	42 (28.6)	
T Stage				0.002
T1–T2	120 (37.4)	75 (43.1)	45 (30.6)	
T3–T4	201 (62.6)	99 (56.9)	102 (69.4)	
N Stage				<0.001
N0	139 (43.3)	88 (50.6)	51 (34.7)	
N+	182 (56.7)	86 (49.4)	96 (65.3)	
M Stage				0.219
M0	312 (97.2)	170 (97.7)	142 (96.6)	
M1	9 (2.8)	4 (2.3)	5 (3.4)	
Overall Stage				<0.001
I–II	94 (29.3)	62 (35.6)	32 (21.8)	
III–IV	227 (70.7)	112 (64.4)	115 (78.2)	
Smoking History				0.014
Never	79 (24.6)	51 (29.3)	28 (19.1)	
Current or former	242 (75.4)	123 (70.7)	119 (80.9)	
Drinking History				0.006
Never	86 (26.8)	56 (32.2)	30 (20.4)	
Current or former	235 (73.2)	118 (67.8)	117 (79.6)	
Betel Nut Use *				—
Available	76 (23.7)	40 (23.0)	36 (24.5)	
Missing	245 (76.3)	134 (77.0)	111 (75.5)	
HPV Status ^†^				—
Available	0 (0)	0 (0)	0 (0)	
Missing	321 (100)	174 (100)	147 (100)	

Data are presented as n (%). Percentages may not sum to 100 due to rounding. * Betel nut-related annotation was available for 76 patients; however, the accessible source field did not distinguish positive from negative exposure status. ^†^ HPV status was not available in the clinical annotations for the TCGA-HNSC-OSCC subset analyzed in this study.

**Table 2:** Primer sequences used for RT-qPCR in this study.

Gene	Primer (5′ to 3′)
ACADL	Forward: CCCGAGCATTTGTGGACAACTG Reverse: GATTGGCTGAACTCTGGCATCC
CAV1	Forward: ACCCCTGCTCAGTAAAGCAC Reverse: ATTACTGCCTCCTCCCCCAT
STC2	Forward: CACTGTTTGGTCAACGCTGG Reverse: AGCGTGGGCCTTACATTTCA
GAPDH	Forward: TGACTTCAACAGCGACACCCA Reverse: CACCCTGTTGCTGTAGCCAAA

## Data Availability

The public datasets analyzed in this study are available from The Cancer Genome Atlas (TCGA) data portal (https://portal.gdc.cancer.gov/ accessed on 2 February 2026) and the Gene Expression Omnibus (GEO) repository (https://www.ncbi.nlm.nih.gov/geo/, accessed on 2 February 2026), reference datasets TCGA-HNSC-OSCC, GSE41613, and GSE172577. The hypoxia- and lipid metabolism-related gene set used is listed in Table S1. The original data generated in this study are included in the article and its Supplementary Materials, including the RT-qPCR raw Ct values and relative expression data in Table S15. Additional information is available from the corresponding authors upon reasonable request.

## Supplementary Materials

The following supporting information can be downloaded at: https://www.mdpi.com/article/10.3390/ijms27104564/s1.

## Abbreviations

The following abbreviations are used in this manuscript:

CAF	cancer-associated fibroblast
CG	candidate genes
DEG	differentially expressed gene
GO	Gene Ontology
HIF	hypoxia-inducible factor
HLMRG	hypoxia- and lipid metabolism-related gene
HNSCC	head and neck squamous cell carcinoma
KEGG	Kyoto Encyclopedia of Genes and Genomes
MDSC	myeloid-derived suppressor cell
OSCC	oral squamous cell carcinoma
TLS	tertiary lymphoid structure
