# Supplementary material for: Integrative Bulk and Single-Cell Transcriptomic Analysis Identifies a Hypoxia- and Lipid Metabolism-Related Prognostic Signature in Oral Squamous Cell Carcinoma: A Retrospective Study
Source: Int J Mol Sci. 2026 May 19;27(10):4564. doi: 10.3390/ijms27104564 (PMC13207035; doi:10.3390/ijms27104564)
Supplement: Supplementary file 1 [file ijms-27-04564-s001.zip › Supplementary Table Notes.pdf]

Table S1 Hypoxia and lipid metabolism-related genes (HLMRGs) acquired from PMID: 39827204.

Table S2 Cell markers acquired from PMID: 34044317.

Table S3 Upregulated and downregulated differentially expressed genes (DEGs) in TCGA-HNSC-OSCC dataset.

Table S4 Candidate genes (CGs) by taking intersection of DEGs and HLMRGs.

Table S5 GO pathways enriched by CGs.

Table S6 KEGG pathways enriched by CGs.

Table S7 Genes passing the PH test in univariate Cox analysis.

Table S8 High-risk and low-risk samples in TCGA-HNSC-OSCC dataset.

Table S9 High-risk and low-risk samples in GSE41613 dataset.

Table S10 Common pathways of prognostic genes.

Table S11 Differential pathways between high-risk and low-risk groups.

Table S12 Correlations between genes and immune cells.

Table S13 Differences of IC_{50} between high-risk and low-risk groups.

Table S14 Correlation between prognostic genes and drugs with differential IC_{50}.

Table S15: RT-qPCR source data.
